# Can the Brain Be Relativistic?

**DOI:** 10.3389/fnins.2021.659860

**Published:** 2021-06-17

**Authors:** Reza Rastmanesh, Matti Pitkänen

**Affiliations:** ^1^Independent Researcher, Private Clinic, Tehran, Iran; ^2^Independent Researcher, Washington, DC, United States; ^3^Independent Researcher, Karkkila, Finland

**Keywords:** human augmentation, human observer, biological physics, quantum physics, speed of light, information processing

Einstein special relativity theory (SRT) suggests that (i) no signal can cause an effect outside the source light cone, the space-time surface on which light rays emanate from the source, and (ii) no signal can move faster than light through a vacuum (Garrison et al., 1998).

There have been some claims that if information processing in the brain relies on nerve pulse conditions alone, the conduction velocities must be relativistic or even super-luminal. This, however, looks highly non-feasible.

Based on a rough estimation, it had been estimated that velocity of neural integration in the cortical paths in the human brain may be close to the speed of light, i.e., to reach 2.28 ×10^8^ *ms*^−1^ that is ~0.76 of light speed (0.76 × *c*) (Ghaderi, 2015). However, equation 7 in that paper (Ghaderi, 2015) extremely over-inflates the numerator (and therefore the velocity) by assuming that neural signals travel sequentially from neuron to neuron. This overestimates the path length by many orders of magnitude.

Theoretically, if by a given selective enhancement it becomes possible to increase the velocity of neural information transfer, i.e., to reach or exceed the speed of light; then some basic laws of physics including the limitations imposed by the speed of light may be violated. Below, using the most updated physiologic and neurophysiologic evidence we will show that the current physics is still right in the view that no signal can cause an effect outside the source light cone.

The brain was claimed to be partially relativistic and obey relativistic principles of general relativity theory (GRT) (Ghaderi, 2015; Le Bihan, 2019). This sparked an enthusiasm that correction of time perception and increasing information exchange velocity by means of selective human augmentation technologies, may—at least in theory—increase the capacity to change the space-time metric perception, and consequently might influence the past-future relationship in the light cone, giving a higher perception and or conception or consciousness to human observers (Migliore et al., 2000).

The velocity of the neural transfer is equal to the distance traveled divided by the time elapsed. So, we should either shorten the distance (i.e., find a shortcut path) or shorten the time elapsed. Remember that implicit to the above-mentioned calculation (Ghaderi, 2015) is that the brain is in a “default mode” with a given activity and information processing which is not 100% of its maximum capacity (Mak et al., 2017; Vatansever et al., 2017), i.e., if, and only if, the activity of cortex is increased, the velocity of information exchange and integration will theoretically increase near to the order of speed of light levels (Ghaderi, 2015).

Studies attempting to map the connectome—the brain's wiring diagram aiming to elucidate the structural and functional connections of the human brain—is suggestive that due to several opportunities including the (i) hyperbolic nature of human brain connectome, i.e., effective hyperbolic geometry in which similar neurons possibly distant in the real geometry are near to each other (Indow, 1997; Cacciola et al., 2017; Seguin et al., 2018; Zhou et al., 2018; Sharpee, 2019; Allard and Serrano, 2020), such as effective hyperbolic geometry in olfactory (Zhou et al., 2018) and visual (Indow, 1997) space, which offers brilliant computational opportunities to find shortcut paths for nanodrugs; and (ii) possibility to increase the speed of information transfer and accurate responses through enhancement of cortex activity via pharmacological drugs (such as Tolcapone which improves cognition and cortical information processing in normal human subjects) (Apud et al., 2007), and (iii) myelinated nature of nerve fibers and existence of Ranvier nodes, which allows an increased rate of action potential transmission due to “jumping” between them, tempts us to theorize that obtaining selective enhancement in sensory systems (visual system acuity, temporal resolution, spatial resolution/acuity, memory acquisition, memory reconsolidation, memory formation, or memory remembrance) may synergically accelerate neural integration, and information transfer velocity. However, the point is that even such a synergism never approaches to the speed of light (Cariani, 2001; He et al., 2014; Sole et al., 2016).

For instance, we know that myelinated axon nerve impulses travel 100 times more rapidly than impulses in non-myelinated axons (Kier and Tombes, 2013). However, due to the nodes of Ranvier, nerve impulses travel as much as 300-fold faster (Kier et al., 2015). This is substantially higher than the default mode, but is still extremely insufficient to reach the speed of light.

It has been proposed that the enhanced action potential passage and flow of information along myelinated axons through the Ranvier node of myelinated axons is carried out *via* a process of *proton hopping* (Kier et al., 2015). Ford et al. (2015) reported unexpected structural specializations in the Ranvier nodes and internodes of auditory brainstem axons involved in sound localization. Myelination properties deviated significantly from the traditionally assumed structure. Axons responding best to low-frequency sounds had a larger diameter than high-frequency axons but, surprisingly, shorter internodes. Simulations predicted that this geometry helps to adjust the conduction velocity and timing of action potentials within the circuit. Electrophysiological recordings *in vitro* and *in vivo* confirmed higher conduction velocities in low-frequency axons. Moreover, internode length decreased and Ranvier node diameter increased progressively along the distal axon segments, which simulations show was essential to ensure precisely timed depolarization of the giant calyx of Held presynaptic terminal. Authors (Ford et al., 2015) conclude individual anatomical parameters of myelinated axons can be tuned to optimize pathways involved in temporal processing.

Some may use above-mentioned findings as a support for their claim. However, even if such a possibility comes true, there is again a huge gap to reach relativistic speeds if purely classical communications by nerve pulses are assumed.

There is also some evidence in support of sensory enhancement:

We first present and then briefly discuss them:

1. It is said that stimulation of a given sensory modality synergizes discrimination of another sensory modality. For instance, visual stimuli significantly influences auditory loudness discrimination (Desantis et al., 2014), auditory sensory positively influences visual temporal rate perception (Recanzone, 2003), olfaction modulates visual perception in a synergistic manner (Zhou et al., 2010) among others. If quantum coherence in brain scale is possible, these effects could be due to the increase of the quantum coherence scale.
2. There is evidence from experimental and clinical trials demonstrating the selective enhancing effect of pharmacological drugs on specific brain areas, suggestive that that every neuron (or group of neurons) embodies different molecular information that hands an operational effect on neuronal computation (Erskine et al., 2004; Rose et al., 2010; Tozzi, 2015; Hagena and Manahan-Vaughan, 2017). For example, in a randomized, double blind, placebo controlled, and crossover design, Apud et al. (2007) evaluated the effects of tolcapone, a central nervous system penetrant specific catecholamine-O-methyltransferase (COMT) inhibitor, on measures of cognitive function and prefrontal cortical information processing in normal subjects stratified by COMT (val158met) genotype. They found significant drug effects on measures of executive function and verbal episodic memory and a significant drug by genotype interaction on the latter, such that individuals with val/val genotypes improved, whereas individuals with met/met genotypes worsened on tolcapone. Functional magnetic resonance imaging (fMRI) revealed a significant tolcapone-induced improvement in the efficiency of information processing in the prefrontal cortex during a working memory test. Their study demonstrated enhancement of prefrontal cortical function in normal human subjects with a non-stimulant drug having COMT inhibitory activity. Since participants were healthy subjects with no family history of psychiatric disorders, with brains at a normal default mode, this study ignited a keen interest that even in normal subjects it is possible to significantly increase information processing capacity and velocity. The above comment about quantum coherence applies here as well.
3. There is also experimental evidence that *time cells* respond selectively to different stimuli (Kraus et al., 2013; Howard et al., 2014; Mau et al., 2018), and *space cells* respond selectively both in an allocentric and egocentric manner (i.e., independent and dependent of reference frames, respectively), in human spatial memory (Woodin and Allport, 1998; Holmes and Sholl, 2005) depending the situation and position. For example, in the absence of vision, decentred egocentric condition involves a frame of reference which seems to be neither allocentric nor totally egocentric (Coluccia et al., 2007). These conjunctive codes for what, when and where are analogous to neurons in the visual system that show a conjunctive code for what, where (Howard, 2018) (and arguably) when.

Apart from technical difficulties, sensory modality synergy is highly implausible to fill huge gap between the neural integration velocity in the cortical paths and speed of light. However, concerning the explanation of the sensory enhancement one may propose several alternative explanations:

a) The increase in nerve pulse conduction velocity could be partially responsible for sensory and cognitive enhancements. Physicists would however argue that it is very implausible that the conduction velocity could approach relativistic speeds. Furthermore, in classical GRT it is in principle impossible to formulate the idea about the increase of light-velocity since there is only single space-time and no reference space-time (Minkowski space-time) with which to compare. In GRT one can however consider wormholes along which the distance would be much shorter than otherwise. The problem of wormholes is that they are not stable. They have been also proposed as correlates of quantum entanglement (ER-EPR correspondence [ER-EPR]) (Maldacena and Susskind, 2013).

b) There is evidence that bio-photons (Bischof, 2005) are relevant for both biology and neuroscience. Popp et al. (1988) and Popp (2003) is one of the pioneers in the study of bio-photons. There is evidence that bio-photon emission from plant leaves is coherent (Bajpai, 1999) and that both DNA (Popp et al., 1984) and cell membrane (Dotta et al., 2011) can act as a source of bio-photons. There is also evidence that bio-photon emission correlates with neural activity (Rahnama et al., 2011), that bio-photon emission correlates with EEG (Sun et al., 2010) and findings suggesting that bio-photons could act as neural communication signals (Persinger et al., 2013). Hence one may argue that bio-photons—or whatever is behind them—propagating along pathways parallel to axons analogous to wave guides could serve as carriers of neuronal and biological information. This would force the views about the role of nerve pulses to be challenged.

Could the neural transmitters in synaptic contacts connect communications lines assignable to axons to longer communications for photons to propagate? There would be analogy with radio communications. Keeping them open all the time could mean too high metabolic energy costs. We remain neutral with regard to claims about bio-photons.

This would also allow feedback to sensory organs as virtual sensory input and hallucinations and rapid eye movement (REM) dreams could be understood in terms of virtual sensory input. The assumption that sensory organs serve as seats of sensory qualia, would allow to get rid of the mystery why essentially identical brain areas can give rise to so different sensory qualia. The basic objection provided by phantom leg could be avoided if the pain in the phantom leg is a sensory memory.

c) If the arrow of time is not universal and could change, apparent superluminal velocities may become possible. The photon traveling to, say, the past and reflected back in the direction of time could return back to the time when it was sent. Could memories be identified as communications with the geometric past of brain: seeing in time direction?

d) Macroscopic quantum coherence and entanglement could make apparent violation of light velocity barrier possible. Whether standard quantum theory is enough to allow macroscopic quantum coherence and cognitive and sensory enhancement is however far from clear. Biosystems are coherent systems, which is extremely difficult to understand in standard quantum theory. Could biology teach something important to physicists?

ER-EPR correspondence claims that wormholes serve as correlates for quantum entanglement in GRT. Could this picture generalize so that the analogs of wormholes (unstable in GRT) would be everyday life in biology?

e) The generation of quantum coherence in brain scale could explain sensory enhancement in a given sensory modality by a stimulus in different modality. The binding of pharmacological drugs to the synaptic contacts could build “bridges” between neurons making quantum coherence in longer scales possible. This coherence would reflect itself as synchronous neuronal firing in larger brain regions.

As can be seen, there are different bottlenecks, wherein the velocity of brain information exchange can be enhanced. However, the sum of these separate enhancements could never reach the speed of light, even if these synergisms are combined. The brain cannot be relativistic.

New physics involving macroscopic quantum coherence and possibly both arrows of time could however allow even instantaneous integration of neuronal information.

## Author Contributions

RR conceptualized the hypothesis of the study and wrote the initial draft of the manuscript. MP critically revised the physics related materials within the paper and contributed to the conception of study. All authors revised the article and participated in the final approval of the manuscript.

## Conflict of Interest

The authors declare that the research was conducted in the absence of any commercial or financial relationships that could be construed as a potential conflict of interest.
